# Work-related stress and intention to leave among midwives working in Swiss maternity hospitals – a cross-sectional study

**DOI:** 10.1186/s12913-021-06706-8

**Published:** 2021-07-08

**Authors:** Karin Anne Peter, Barbara Meier-Kaeppeli, Jessica Pehlke-Milde, Susanne Grylka-Baeschlin

**Affiliations:** 1 Division of Applied Research & Development in Nursing, Bern University of Applied Sciences, Murtenstrasse 10, 3008 Bern, Switzerland; 2 Division of Women’s Health and Newborn Care, Department of Obstetrics, University Hospital of Zurich, Frauenklinikstrasse 10, CH-8091 Zürich, Switzerland; 3 Research Unit for Midwifery Science, ZHAW Zurich University of Applied Sciences, Katharina-Sulzer-Platz 9, CH-8401 Winterthur, Switzerland

**Keywords:** Midwifery, Stressors, Intention to leave, Job satisfaction, Work force retention

## Abstract

**Background:**

Health systems around the globe are struggling to recruit qualified health professionals. Work-related stress plays an important role in why health professionals leave their profession prematurely. However, little is known about midwives’ working conditions and intentions to leave their profession, although this knowledge is key to work force retention. Therefore, we aimed to investigate work-related stress among midwives working in Swiss maternity hospitals, as well as differences between midwives and other health professionals and the stressors associated with midwives’ intention to leave the profession.

**Methods:**

We conducted a data analysis of two cross-sectional studies encompassing midwives working in labour, postpartum and/or gynaecology wards of 12 public Swiss maternity hospitals. Data was collected by self-report questionnaire assessing potential stressors and long-term consequences of stress at work. Data were analysed using descriptive statistics, Kruskal Wallis tests and logistic regression modelling.

**Results:**

A total of 98 midwives took part in the study and one in three midwives reported doing overtime sometimes-always. Also, the score for work-private life conflicts was significantly higher among midwives than among other health professionals, with the exception of physicians (M = 37.0 versus 50.2, *p* < .001). Midwives’ meaning of work score (M = 89.4) was significantly higher than that of other health professionals (e.g. nurses (M = 83.0, *p* < .001) or physicians (M = 82.5, *p* < .01)). Generation Y midwives showed a significantly higher intention to leave their organisation than did the baby boomers (Mean scores 29.3 versus 10.0, *p* < .01). Results of the regression model revealed that if midwives could compensate for their overtime in the same month, their intention to leave the profession was lower (OR = 0.23, *p* < .05). Additionally, the more midwives were affected by work-private life conflicts (OR = 3.01, *p* < .05) and thoughts about leaving their organisation (OR = 6.81, *p* < .05), the higher was their intention to leave their profession prematurely.

**Conclusions:**

The comparison with other health professions and the higher intention to leave the profession of younger midwife generations are important findings for heads of institutions as well as policy makers, and should stimulate them to develop strategies for keeping midwives on their staff. More extensive studies should implement and test interventions for reducing work-related stress and increasing the job and occupational satisfaction of midwives.

## Background

Health systems are struggling with the workforce shortage and with recruiting qualified health professionals around the globe [1]. The Word Health Organization WHO emphasises the need for a healthcare force of adequate size and skills to ensure well-functioning health care systems [2]. Among healthcare employees, over 40 % of midwives, nurses and medical-technical professionals are the most likely to leave their profession prematurely [3].

Stress at work, poor working conditions and reward frustration are associated with health professionals’ higher intention to leave their profession prematurely [4, 5]. Midwives and nurses are especially affected by work-related stress, due to higher emotional demands at work and lower opportunities for development compared to other health professionals (e.g. physicians, medical-technical and medical-therapeutic professionals) [6]. Also, other authors have identified high workload, lack of appreciation by doctors, interruptions of work, high working hours, unclear job roles, difficulties with emotional demarcation as well as the incompatibility of work and family life as important stressors among midwives. Moreover, some of these stressors (e.g. work interruptions, high workload) seemed to be more strongly pronounced among midwives working in acute care hospitals, than those working in out-of-hospital care [7]. Finally, a previous study revealed that midwives and nurses have the lowest job satisfaction and the highest intention to leave amongst all health professionals [6].

An integrative review by Bloxsome [8] et al. identified seven themes that were related to the job satisfaction of midwives and their intention to stay in the profession: relationships with colleagues, relationships with women clients, enjoying the job and being proud to be a midwife, liking to care for women, good hours and money, passion for midwifery and enjoying the variety in the work. Furthermore, a recent study in Germany including more than 2,000 midwives working in hospitals showed that 42 % of midwives were not satisfied or not at all satisfied with recognition of their work, while 33 % were similarly dissatisfied with the compatibility of their work with family life and 39 % with their general working conditions [9]. Additionally, the authors found that nearly half of the midwives (48 %) were dissatisfied with the possibility to reduce overtime. As a consequence of their dissatisfaction, 22 % of the midwives stated that is was unlikely or rather unlikely that they would choose the profession again.

The European commission recognises that the capacity to maintain health professionals in the health care system plays a major role in strategies to prevent workforce shortage [10]. However, to retain health professionals and prolong their stay in the profession, it is important to address work-related stressors, thus improving working conditions [11]. Switzerland is, among other countries, also struggling with a shortage of nurses and midwives [3]. Furthermore, working conditions differ from those in other European countries (e.g., Swiss employees have a higher number of working hours per week in full-time employment) [12]. Studies focusing on nurses’ working conditions and intention to leave have already been conducted for Switzerland [13, 14]. However, in these studies, midwives have often been grouped with nurses in the analysis, so that no conclusions can be drawn about the working conditions of midwives as a specific group [4, 6]. Hence, studies with a main focus on midwives’ individual conditions at work and important stressors triggering their intention to leave the profession prematurely are needed in order to develop appropriate prevention and intervention strategies.

Therefore, the aim of this study is to investigate (a) the compatibility of work and private life among midwives working in Swiss maternity hospitals, to identify differences between (b) midwives and other health professionals and between (c) different midwife generations regarding work-related stress as well as d) stressors associated with midwives’ intention to leave the profession.

## Methods

### Design

**Fig. 1:**
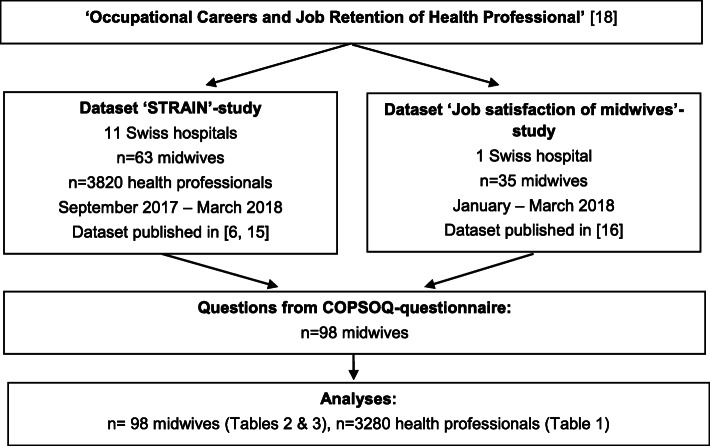
Flowchart explaining datasets included for this study

This study is based on a cross-sectional design, using secondary data from the national STRAIN study - ‘work-related **STR**ess **A**mong health professionals **IN** Switzerland’ [6, 15] and the study ‘Job satisfaction of midwives’, part of the project ‘Occupational Careers and Job Retention of Health Professionals’ [16, 17]. Both studies were part of a cooperation between the Swiss Universities of Health to establish a competence centre for workforce shortage among health professionals [18]. Different sub-projects developed basic knowledge and measures. Data was collected from September 2017 until March 2018 in Swiss public maternity hospitals using midwives’ self-reports on stress at work and included n = 98 midwives and n = 3820 other health professionals from the STRAIN study [6] (see Fig. 1).

### Recruitment and data collection

The Swiss health care system is based on aspects of managed competition and influences of direct democracy, encompassing 293 hospitals that vary in size from 2 to 2000 beds [19]. These hospitals are divided into acute care, rehabilitation and psychiatric hospitals.

For the STRAIN study, acute care hospitals with a minimum average of 20 beds or 7 employees were randomly selected (using randomizer.org) from a list of all registered acute care hospitals in Switzerland in 2016 and invited to participate. The midwife sample of the STRAIN study was collected in 11 maternity hospitals. Data was collected using a contact person in each hospital, who distributed the questionnaires via email or in a printed-paper version as well as giving information about the study to all midwives at all skill levels. Further information on the entire recruitment process for the total ‘STRAIN’ dataset that included health professionals working in Swiss hospitals (n = 3280) are published in Peter, et al. [6].

The ‘Job satisfaction of midwives study’ was conducted in a selected Swiss public hospital. Data from the study ‘Job satisfaction midwives’ was collected with midwives working in the labour ward as part of a practice project before the implementation of a midwife-led intervention was used for baseline values.

In the end, 98 midwives from 12 maternity hospitals (11 from the STRAIN, one from the ‘Job satisfaction of midwives’ study) could be included in our data analysis.

### Questionnaire

For this study the STRAIN questionnaire [6, 20] and a questionnaire designed to assess the occupational satisfaction of midwives [16] including questions from the STRAIN survey were used. Both questionnaires focused on potential stressors at work as well as possible long-term consequences on employees, and included the following valid and reliable self-assessment scales or single items from the Copenhagen Psychosocial Questionnaire – COPSOQ [21–23] on the ‘work-privacy conflict’, i.e. the conflict between work and private life (5 items on a five-point Likert scale), difficulties with ‘demarcation’, e.g. being available for work issues during leisure time (2 items), ‘meaning of work’, e.g. perceiving work as meaningful or important (2 items), ‘bond with the organisation’, e.g. being proud to belong to this organisation (2 items), ‘job satisfaction’, e.g. being pleased with work prospects, conditions (6 items), ‘intention to leave the organisation’. e.g. thoughts on job changes (single item) and ‘intention to leave the profession’, e.g. thoughts on career change (single item). Response options for all items were on a five-point Likert scale. In addition, demographic data (e.g. age, gender, educational level) were collected as well as additional questions on overtime performance. The questionnaire was available in German, French and Italian.

### Data analysis

According to the original author of the German COPSOQ Version [21], all items based on the COPSOQ were transformed to a value range extending from 0 (minimum value) to 100 points (maximum value). This transformation is necessary to make the results of this study comparable with other studies. No average score was calculated if fewer than half of the questions in a scale had been answered, following Kristensen [22, 23].

First, descriptive statistics on single items regarding overtime and possibilities for compensation of overtime were calculated. Second, to test for significant differences between midwives and other health professionals (nurses, physicians, medical-technical and medical-therapeutic professionals) the dataset of this study (midwives only) was compared with the dataset of the STRAIN study including other health professionals. Also, significant differences regarding various midwife generations (generation baby boomer, generation X, generation Y) were calculated. Therefore, the independent-samples Kruskal-Wallis test using pairwise comparison (testing the null hypothesis that the two compared samples’ distributions are the same) adjusted by the Bonferroni correction for multiple test (adjusted significance, 2-sided test at the level of 0.05) was used to test for significant differences between midwives and other health professionals. The Kruskal-Wallis test was used since there were no equal-sized samples of data.

**Fig. 2:**
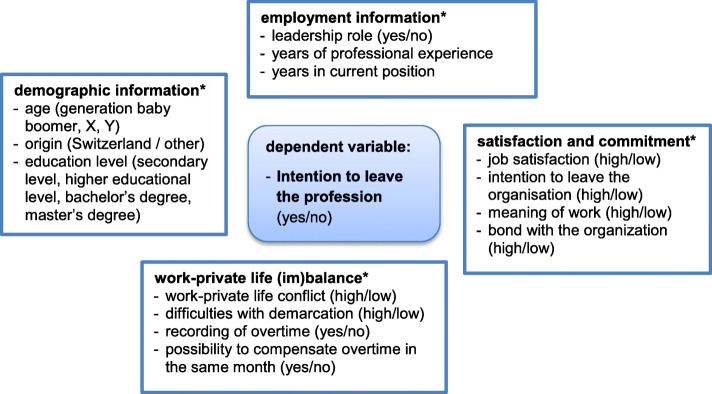
dependent and independent variables in the logistic regression model . *variables used as ‘independent variables’ in the regression models.

Third, we estimated a logistic regression model for the outcome variable ‘intention to leave the profession’ (see Fig. 2). Therefore, all variables included in the regression model presented in Fig. 1 were dummy-coded (yes/no or higher/lower than median) or included as metric variables (e.g. years of professional experience / in the current position). For the logistic regression model, all independent variables were included and selected using the stepwise backward algorithm (R-package MASS, function stepAIC) and the Akaike Information Criterion (AIC). To test for multicollinearity between the regression coefficients, the variance inflation factor (VIF) was used. In addition, the explained variance (R square), the exponentiation of the beta coefficient, beta coefficient, standard error, z-values and p-values (2-tailed) were calculated. Since the assumption of homoscedasticity was not met, p-values were computed based on bootstrap (r = 1000 bootstrap, bias corrected and accelerated, 95 % CI). Data was analysed using R 3.6.0.

## Results

### Study sample

In total, 98 midwives working in public maternity hospitals took part in the study (overall response rate of participants was 64 %; STRAIN study = 43 %, Job satisfaction of midwives = 85 %). Of these, 95 % were German-speaking and 5 % French-speaking. All participants were female, and between 23 and 64 years old (mean = 40, SD = 12). Most participants (79 %) were born in Switzerland, 11 % in Germany, 2 % in France and 8 % in other countries (e.g. Serbia, Italy). Most participants were highly educated (66 % had a bachelor’s or master’s degree) and had no management responsibility at work (89 %), while 11 % of them worked in a management position. Participants had between 2 and 43 years of professional experience (mean = 18, SD = 12) and had worked between a few months and 39 years in their current position (mean = 10, SD = 10).

### Descriptive results on overtime and work-private life conflicts

Most midwives reported that they had to do overtime ‘sometimes’ (39 %) or ‘often’ to ‘always’ (30 %). Almost all of them (98 %) were allowed to record their overtime while 2 % stated that they had no means with which to record it. Most midwives reported that it was possible to compensate for their overtime in the same (31 %) or in the following month (81 %), while for 18 % of midwives it was possible to receive supplementary payment for their overtime and 5 % stated that it was impossible to be compensated for their overtime (either by time off or supplementary payment).

### Differences between midwives and other healthcare professionals

Regarding differences between midwives and other health professionals, shown in Table 1, results of the Kruskal-Wallis test revealed that midwives (mean(M) = 89.4, SD = 12.8) reported a slightly but significantly higher meaning of work than did nurses (M = 83.0, SD = 15.9, *p* < .001), physicians (M = 82.5, SD = 18.2, *p* < .01), medical-technical (M = 84.0, SD = 16.2, *p* < .01) and medical-therapeutic professionals (M = 79.7, SD = 15.7, *p* < .001). For the scale on bond with the organisation, no significant differences (*p* > .05) between midwives and other health professionals was found. Further results revealed more work-private life conflicts among midwives (M = 37.0, SD = 17.4) than among nurses (M = 33.1, SD = 21.4, *p* < .05), medical-technical (M = 25.5, SD = 18.7, *p* < .001) and medical-therapeutic professionals (M = 23.0, SD = 17.8, *p* < .001), but less work-private life conflicts than among physicians (M = 50.2, SD = 22.2, *p* < .001). Midwives reported having fewer problems with demarcation (M = 32.5, SD = 20.2) compared with physicians (M = 49.6, SD = 25.1, *p* < .001), but no further significant differences regarding problems with demarcation were found between midwives and other health professionals. Regarding midwives’ job satisfaction (M = 68.4, SD = 11.0), results revealed lower job satisfaction among midwives compared with physicians (M = 72.1, SD = 15.5, *p* < .05) and medical-therapeutic professionals (M = 71.6, SD = 13.8, *p* < .05). In addition, midwives’ intention to leave the organisation was significantly higher (M = 23.4, SD = 22.5) compared with medical-technical professionals (M = 18.9, SD = 22.9, *p* < .05), but not with other health professionals (*p* > .05). Further results on midwives’ intention to leave the profession also revealed higher intentions among midwives (M = 17.0, SD = 20.2) compared with medical-technical professionals (M = 12.9, SD = 20.7, *p* < .05), but were not significantly different from other health professionals (*p* > .05).

**Table 1:** Differences regarding work-related stress between midwives and other healthcare professionals

	midwives^**1**^			all health prof.			nursing staff^2^			K-W test	physicians^3^			K-W test	medical-technical prof.^4^			K-W test	medical-therapeutic prof.^5^			K-W test
	*n*=98			*n*=3280			*n*=1806				*n*=294				*n*=215				*n*=225			
	Mdn	M	SD	Mdn	M	SD	Mdn	M	SD	1 vs. 2	Mdn	M	SD	1 vs. 3	Mdn	M	SD	1 vs. 4	Mdn	M	SD	1 vs. 5
**meaning of work**	100	89.4	12.8	87.5	82.6	16.4	87.5	83.0	15.9	3.9***	87.5	82.5	18.2	3.3**	87.5	84.0	16.2	2.9**	75	79.7	15.7	5.3***
**bond with the organisation**	62.5	59.4	17.5	62.5	59.7	20.1	62.5	57.9	20.1	.9	62.5	59.9	19.0	.0	62.5	63.8	18.4	1.7	62.5	60.4	17.8	.2
**work – privacy conflict**	37.5	37.0	17.4	30	32.3	21.8	30	33.1	21.4	2.2*	50	50.2	22.2	4.2***	25	25.5	18.7	4.9***	20	23.0	17.8	5.9***
**demarcation**	25	32.5	20.2	37.5	34.9	22.2	37.5	33.3	21.1	.3	50	49.6	25.1	5.7***	37.5	36.9	22.3	1.6	25	31.9	21.7	.2
**job satisfaction**	70.8	68.4	11.0	70.8	68.8	14.9	70.8	67.6	14.	.3	70.8	72.1	15.5	2.2*	70.8	69.7	14.0	.9	70.8	71.6	13.8	2.2*
**intention to leave the organisation**	25	23.4	22.5	25	22.3	23.5	25	23.0	23.1	.3	25	24.3	24.8	.1	25	18.9	22.9	2.1*	25	21.2	22.7	.8
**intention to leave the profession**	12.5	17.0	20.2	0	17.3	22.3	25	18.9	22.5	.7	0	15.8	21.3	.6	0	12.9	20.7	2.2*	0	12.7	19.2	1.9

1 = midwives, 2 = nursing staff, 3 = physicians, 4 = medical-technical professionals, 5 = medical-therapeutic professionals, all scales are scored from 0 (minimum value) to 100 (maximum value). *n*= number of cases, *Mdn* Median, *M* mean, *SD* standard deviation, standardised test statistics of Kruskal-Wallis test adjusted by the Bonferroni correction for multiple tests between midwives and other healthcare professionals at the level *p ≤ .05; **p < .01; ***p < .001

### Results regarding different generations

In Table 2, significant differences revealed by the Kruskal-Wallis test are presented for the Baby-Boomer generation (1946–1964), generation X (1965–1979) and generation Y (1980–1993). The results showed a significant difference for intention to leave the organisation between members of the baby boomer generation (M = 10.0, SD = 15.8) and those of generation Y (M = 29.3, SD = 22.5, p < .01). However, for meaning of work, bond with the organisation, work-private life conflicts, difficulties with demarcation and intention to leave the profession prematurely, no significant differences (p < .05) between generations baby boomer, X and Y were identified.

**Table 2:** Differences regarding work-related stress between various midwife-generations

	generation baby boomer^1^			generation X^2^			generation Y^3^			K-W test			
	*n*=15			*n*=34			*n*=48			*n*=96			pairwise comparisons
	Mdn	M	SD	Mdn	M	SD	Mdn	M	SD	test	DF	sign.	
**meaning of work**	87.5	89.2	11.4	93.8	89.0	12.2	100	89.9	13.9	.4	2	.806	
**bond with the organisation**	50.0	59.2	19.2	56.3	59.4	13.8	62.5	59.2	19.4	.2	2	.901	
**work-private life conflicts**	25.0	31.2	18.8	41.9	38.9	17.5	36.3	37.9	17.0	2.7	2	.264	
**demarcation**	25.0	25.0	22.2	25.0	32.8	20.5	37.5	34.4	19.1	2.5	2	.292	
**job satisfaction**	70.8	69.2	7.5	72.9	70.2	10.5	68.8	66.8	12.2	2.1	2	.350	
**intention to leave the organisation**	0.0	10.0	15.8	25.0	21.9	22.7	25.0	29.3	22.5	9.5	2	**.009**	1 vs. 3=22.8**
**intention to leave the profession**	0.0	11.7	16.0	25.0	18.8	21.1	12.5	17.4	21.0	1.2	2	.561	

1 = generation baby-boomer (1946-1964), 2 = generation X (1965-1979), 3 = generation Y (1980-1993), all scales are scored from 0 (minimum value) to 100 (maximum value). *n* number of cases, *Mdn* Median, *M* mean, *SD* standard deviation, standardised test statistics of Kruskal-Wallis test adjusted by the Bonferroni correction for multiple tests between Generations at the level *p ≤ .05; **p < .01; ***p < .001

### Results of the logistic regression on the intention to leave the profession

Results from the final logistic model on the intention to leave the profession are presented in Table 3. The selected predictors using AIC backwards selection explained 31.2 % of the total variance. The results revealed that if midwives can be compensated for their overtime in the same month with vacations or free hours / days, their intention to leave the profession is significantly lower (OR = 0.23, p < .05). In addition, the more midwives were affected by work-private life conflicts, the higher was their intention to leave their profession prematurely (OR = 3.01, p < .05). Further results imply that midwives who think about leaving their organisation more frequently also have a higher intention to leave their profession prematurely (OR = 6.81, p < .05).

**Table 3:** Results of the logistic regression model on midwives’ intention to leave the organisation

independent variables	Odds-ratio Exp(B)	Log-Odds (B)	stand. error	z-value	p-value^1^	VIF
intercept	0.13	-2.03	0.63	-3.22		
years in current position	1.06	0.06	0.03	2.00	0.052	1.10
compensation of overtime	0.23	-1.47	0.73	-2.01	0.044*	1.15
bond with the organisation	2.15	0.76	0.53	1.43	0.171	1.05
work-private life conflict	3.01	1.10	0.54	2.03	0.049*	1.10
difficulties with demarcation	2.21	0.79	0.55	1.50	0.166	1.09
intention to leave the organisation	6.81	1.92	0.66	2.88	0.005**	1.13

Independent variables = coefficients, *exp. B* exponentiation of the B coefficient, stand. *error* standard error, ^1^*p*-values were computed based on bootstrap (*r* = 1000 bootstrap, bias corrected and accelerated, 95 % CI), *VIF* variance inflation factor, *p < .05. **p < .01. ***p < .001

## Discussion

This study provides, for the first time, important information on work-related stress among Swiss midwives and on differences from those among other health professionals. Meaning of work and work-private life conflicts were significantly higher in midwives compared with nursing, medical-technical and medical-therapeutic staff and, in the case of the meaning of work, also higher than physicians. Additionally, the intention to leave their healthcare organisation was higher for the younger generations X and Y than for the older baby boomers. Also, midwives who could not compensate for their overtime within the same month and/or were more affected by work-private life conflicts showed a higher intention to leave their profession prematurely. The intention to leave the profession was also higher in midwives who intended to leave the institution.

The midwives in our study reported a slightly higher meaning of work than did nurses, physicians, medical-technical and medical-therapeutic professionals. Even though the difference found was small, meaning of work seems important for job and occupational satisfaction and for work retention, because Bloxsome et al. [8] reported passion for midwifery as an important factor in overcoming row days and staying in the profession. Furthermore, Bloxsome and Bayes [24] in a qualitative study in Australia, concluded that the preference for being with women and the feeling of being able to make a difference to them is an important factor which should inform work policies. Person-centred work was also very important for the meaning of work of nurses working in nursing homes [25]. It might be that maternity care is associated with more individual as well as one-to-one care than other health care, and that midwives therefore have more opportunities for the provision of woman-centred care. This might be an explanation for why the meaning of work is higher in midwives than in other health professionals, but further research is necessary to fully understand the phenomenon.

The current study showed that midwives working in acute care hospitals had more work-private life conflicts than did nurses, medical-technical and medical-therapeutic professionals. These results are in line with a previous results from the STRAIN study, in which midwives revealed higher mean scores regarding work-private life conflicts compared to nurses, medical-technical and medical-therapeutic professionals [26]. However, studies investigating job satisfaction and work-related stressors of midwives showed that the impact of work on the private life of midwives working outside of hospital care was significantly higher than those working in hospitals [7, 27]. It can therefore be assumed that the difference between midwives working in out-of-hospital care and those of other health professionals would be even higher. However, midwives working in out-of-hospital care perceived lower psychological stress and higher levels of autonomy than those working in hospitals [28, 29]. They were also able to provide midwife-led continuity of care which was found to be associated with higher levels of job satisfaction [30]. The higher work-private life conflict of midwives working in out-of-hospital care might be balanced by other stressors that are lower than for midwives working in hospitals and with a better work environment.

On the other hand, our results revealed that younger generations (generation Y / millennials) had a higher intention to leave the organisation. As some authors have suggested [31, 32] younger generations have different needs, aspirations and expectations from those who entered the labour marked before them. Therefore, organisational cultures and strategies suited to their needs are essential in order to retain younger midwife generations. These younger midwives also have more remaining working years, which means the effort might be doubly rewarded. Further research is needed to help develop policies and interventions to reduce work-private life conflicts and determine the impact of such interventions on job satisfaction and work retention.

Albrecht et al. [9] showed that German midwives working in hospitals were very unsatisfied with the opportunity to reduce overtime and Cronie et al. [27] found that working hours were an important factor in the job satisfaction of Dutch midwives working in hospitals. Additionally, several studies with nurses showed that those who worked overtime had a stronger intention to leave the profession [33, 34]. The finding of our study that the possibility to compensate for overtime during the same month was associated with a weaker intention to leave their profession prematurely added the insight that the crucial point might not be the overtime itself but the opportunity to compensate it quickly. This should be considered in work scheduling. Additionally, previous studies showed that work-private life conflict was associated with job satisfaction [7, 27]. The current study added the independent relation of work-private life conflict with intention to leave the profession. This is also an important factor to be considered when developing strategies to support work retention.

### Strengths and limitations

One strength of our study was its multicentre character and the randomised sample. The results reflect the work-related stress of midwives working in twelve maternity hospitals, which minimises the risk that its only related to structural- instead of profession-related factors. An additional strength was the opportunity to compare the work-related stressors of midwives with those of other health professionals, since the same questionnaire was used. On the other hand, the sample size of the study was rather small what might impair the generalisability of results. It is possible that a larger sample of midwives would have revealed more significant predictors in the regression model. Also, the study used a cross-sectional design, which means that causal conclusions cannot be drawn from its results. In addition, participation was voluntary for all midwives and a selection bias is, therefore, possible (e.g. midwives with a higher workload might not have participated for reasons of time).

## Conclusions

Working as a midwife was shown to mean a lot to the participants of this study; nevertheless, this very meaningful work was often related to high levels of work-private life conflict. This was especially true for younger generations. Furthermore, work-private life conflict and not being able to compensate for overtime in the near future was found to be associated with a stronger intention to leave the profession prematurely. In order to be able to keep midwives in their profession, effective strategies to enhance the compatibility of their work with private life seems essential. In addition, a special focus on the needs of younger midwife generations are important. These findings are of importance for policy makers, heads of institutions and also heads of labour wards. Midwives working in institutions are on a lower hierarchical level and depend on the planning and goodwill of their superiors. Additionally, working conditions also depend on staff resources. Staff shortages lead to lower possibilities to re-arrange work schedules to suit the personal needs of employees and thus lower the chances of compensating for overtime within a short period. This, in turn, feeds into a vicious circle, with more midwives quitting their job and profession with increased workforce shortages. Therefore, further research is needed with larger samples and different working environments, e.g. midwives in out-of-hospital settings, to fully understand midwife-specific, work-related stressors. Additionally, such studies should implement and test interventions for reducing work-related stress and increasing the job and occupational satisfaction of midwives.

## Data Availability

The raw dataset analysed in the current study is available from the corresponding author (karin.peter@bfh.ch) on reasonable request.

## Abbreviations

AIC: Akaike Information Criterion
COPSOQ: Copenhagen Psychosocial Questionnaire
STRAIN: work-related stress among health professionals in Switzerland
